# Effectiveness of Bivalent mRNA COVID-19 Vaccines in Preventing COVID-19–Related Thromboembolic Events Among Medicare Enrollees Aged ≥65 Years and Those with End Stage Renal Disease — United States, September 2022–March 2023

**DOI:** 10.15585/mmwr.mm7301a4

**Published:** 2024-01-11

**Authors:** Amanda B. Payne, Shannon Novosad, Ryan E. Wiegand, Morgan Najdowski, Danica J. Gomes, Megan Wallace, Jeffrey A. Kelman, Heng-Ming Sung, Yue Zhang, Bradley Lufkin, Yoganand Chillarige, Ruth Link-Gelles

**Affiliations:** ^1^Coronavirus and Other Respiratory Viruses Division, National Center for Immunization and Respiratory Diseases, CDC, ^2^Division of Healthcare Quality Promotion, National Center for Emerging and Zoonotic Infectious Diseases, CDC; ^3^Centers for Medicare & Medicaid Services, Baltimore, Maryland; ^4^Acumen LLC, Burlingame, California.

**Summary**

**What is already known about this topic?**

Thromboembolic complications of COVID-19 include ischemic stroke, venous thromboembolism, and myocardial infarction. COVID-19 vaccines are effective in preventing severe outcomes, including hospitalization and death.

**What is added by this report?**

During September 2022–March 2023, receipt of bivalent mRNA COVID-19 vaccine was 47% effective in preventing thromboembolic events among immunocompetent persons aged ≥65 years and 51% effective among adults aged ≥18 years with end stage renal disease (ESRD) receiving dialysis, compared with receipt of the original monovalent vaccines alone.

**What are the implications for public health practice?**

COVID-19 vaccines helped provide protection against COVID-19–related thromboembolic events. Persons aged ≥65 years and adults with ESRD should receive all recommended COVID-19 vaccine doses to prevent COVID-19–associated complications, including thromboembolic events.

## Abstract

COVID-19 has been associated with an increased risk for thromboembolic events, including ischemic stroke, venous thromboembolism, and myocardial infarction. Studies have reported lower rates of COVID-19–related thromboembolic events among persons who received the COVID-19 vaccine compared with persons who did not, but rigorous estimates of vaccine effectiveness (VE) in preventing COVID-19–related thromboembolic events are lacking. This analysis estimated the incremental benefit of receipt of a bivalent mRNA COVID-19 vaccine after receiving an original monovalent COVID-19 vaccine. To estimate VE of a bivalent mRNA COVID-19 dose in preventing thromboembolic events compared with original monovalent COVID-19 vaccine doses only, two retrospective cohort studies were conducted among Medicare fee-for-service enrollees during September 4, 2022–March 4, 2023. Effectiveness of a bivalent COVID-19 vaccine dose against COVID-19–related thromboembolic events compared with that of original vaccine alone was 47% (95% CI = 45%–49%) among Medicare enrollees aged ≥65 years and 51% (95% CI = 39%–60%) among adults aged ≥18 years with end stage renal disease receiving dialysis. VE was similar among Medicare beneficiaries with immunocompromise: 46% (95% CI = 42%–49%) among adults aged ≥65 years and 45% (95% CI = 24%–60%) among those aged ≥18 years with end stage renal disease. To help prevent complications of COVID-19, including thromboembolic events, adults should stay up to date with COVID-19 vaccination.

## Introduction

Complications of COVID-19 include an increased risk for thromboembolic events, including ischemic stroke, venous thromboembolism, and myocardial infarction (*1*). Adults aged ≥65 years and persons with end stage renal disease (ESRD) receiving dialysis are at increased risk for thromboembolic events, including COVID-19–related thromboembolic events (*2*). COVID-19 vaccination has been shown to be protective against severe COVID-19–associated outcomes, including hospitalization, mechanical ventilation, and death (*3*,*4*). In addition, rates of COVID-19–related thromboembolic events have been reported to be lower among vaccinated persons than among unvaccinated persons (*5*); however, rigorous estimates of COVID-19 vaccine effectiveness (VE) in preventing COVID-19–related thromboembolic events are not available. This analysis aimed to assess relative effectiveness of bivalent COVID-19 mRNA vaccines compared with original monovalent COVID-19 vaccines alone against COVID-19–related thromboembolic events, stratified by time since dose, among Medicare fee-for-service beneficiaries aged ≥65 years and among those aged ≥18 years with ERSD receiving dialysis.

## Methods

Two retrospective cohort studies were conducted, one among Medicare fee-for-service beneficiaries aged ≥65 years and one among Medicare beneficiaries aged ≥18 years with ESRD receiving dialysis.* Medicare Parts A and B enrollment and claims records were used to obtain information on study participation eligibility,^†^ COVID-19 vaccination status,^§^ covariates,^¶^ and outcomes.** Beneficiaries included^††^ in this study were eligible to receive the bivalent COVID-19 mRNA vaccine.^§§^ All beneficiaries entered the study cohorts on September 4, 2022 (the index date); vaccination status was updated daily, and beneficiaries began contributing time to the bivalent vaccine cohorts beginning 7 days after receipt of a bivalent vaccine dose. Follow-up continued until the earliest occurrence of a censoring event,^¶¶^ study end (March 4, 2023), or COVID-19–related thromboembolic event (ischemic stroke, venous thromboembolism, or myocardial infarction from 7 days before through 30 days after COVID–19 diagnosis). A marginal structural Cox model*** was used to estimate relative VE,^†††^ which can be interpreted as the incremental benefit of a bivalent dose compared with only the original monovalent vaccine doses without a bivalent dose, by immunocompromise status^§§§^ and time since vaccination. Two-sided 95% CIs were calculated for each VE estimate, with 95% CIs that excluded zero considered statistically significant. Nonoverlapping CIs were interpreted as statistically significantly different effectiveness estimates. This activity was reviewed by CDC, deemed not research, and was conducted consistent with applicable federal law and CDC policy.^¶¶¶^

## Results

### Bivalent Vaccine Coverage

During September 4, 2022–March 4, 2023, among 12,706,176 immunocompetent Medicare beneficiaries aged ≥65 years who had previously received an original COVID–19 vaccine, 5,683,208 (44.7%) received a bivalent dose (Table 1). Overall, higher percentages of bivalent vaccine recipients than nonrecipients resided in an urban area (83% versus 78%), had received an influenza vaccine during the 2021–22 season (82% versus 55%) and 2022–23 season (87% versus 50%), and had received an original monovalent booster vaccine dose (96% versus 73%).

**TABLE 1:** Characteristics of immunocompetent Medicare fee-for-service beneficiaries aged ≥65 years and beneficiaries aged ≥18 years with end stage renal disease receiving dialysis* without additional immunocompromising conditions, by receipt of bivalent mRNA COVID-19 vaccine — United States, September 2022–March 2023

Characteristic	Beneficiaries aged ≥65 yrs (N = 12,706,176)				Beneficiaries aged ≥18 yrs with ESRD receiving dialysis (N = 78,618)			
	Overall no. (column %)	Original vaccine only^†^ No. (column %)	Bivalent vaccine^§^ No. (column %)	SMD^¶^	Overall no. (column %)	Original vaccine only No. (column %)	Bivalent vaccine No. (column %)	SMD
**Age group, yrs**								
18–64	**—**	**—**	**—**	**—**	30,240 (38.5)	23,001 (41.5)	7,239 (31.2)	0.22
≥65	12,706,176 (100)	7,022,968 (100)	5,683,208 (100)	**—**	48,378 (61.5)	32,388 (58.5)	15,990 (68.8)	0.22
**Sex**								
Men	5,324,511 (41.9)	2,940,150 (41.9)	2,384,361 (42.0)	0	45,347 (57.7)	31,751 (57.3)	13,596 (58.5)	0.02
Women	7,381,665 (58.1)	4,082,818 (58.1)	3,298,847 (58.0)	0	33,271 (42.3)	23,638 (42.7)	9,633 (41.5)	0.02
**Race and ethnicity**								
Asian or Pacific Islander, NH	299,359 (2.4)	175,013 (2.5)	124,346 (2.2)	0.02	4,647 (5.9)	3,269 (5.9)	1,378 (5.9)	0
Black or African American, NH	639,186 (5.0)	404,907 (5.8)	234,279 (4.1)	0.08	22,731 (28.9)	16,698 (30.1)	6,033 (26.0)	0.09
White, NH	10,971,824 (86.4)	6,007,977 (85.5)	4,963,847 (87.3)	0.05	38,226 (48.6)	25,857 (46.7)	12,369 (53.2)	0.13
Hispanic or Latino	136,672 (1.1)	103,341 (1.5)	33,331 (0.6)	0.09	6,729 (8.6)	5,220 (9.4)	1,509 (6.5)	0.11
Other, NH	659,135 (5.2)	331,730 (4.7)	327,405 (5.8)	0.05	6,285 (8.0)	4,345 (7.8)	1,940 (8.4)	0.02
Social Vulnerability Index ranking**								
≥1 to ≤10	1,426,615 (11.2)	678,323 (9.7)	748,292 (13.2)	0.11	3,793 (4.8)	2,362 (4.3)	1,431 (6.2)	0.09
>10 to ≤20	1,534,203 (12.1)	765,545 (10.9)	768,658 (13.5)	0.08	4,979 (6.3)	3,205 (5.8)	1,774 (7.6)	0.07
>20 to ≤30	1,601,416 (12.6)	820,302 (11.7)	781,114 (13.7)	0.06	5,942 (7.6)	3,854 (7.0)	2,088 (9.0)	0.08
>30 to ≤40	1,502,011 (11.8)	804,184 (11.5)	697,827 (12.3)	0.03	6,741 (8.6)	4,585 (8.3)	2,156 (9.3)	0.04
>40 to ≤50	1,411,576 (11.1)	782,312 (11.1)	629,264 (11.1)	0	7,149 (9.1)	4,850 (8.8)	2,299 (9.9)	0.04
>50 to ≤60	1,265,748 (10.0)	721,828 (10.3)	543,920 (9.6)	0.02	7,308 (9.3)	5,070 (9.2)	2,238 (9.6)	0.02
>60 to ≤70	1,275,330 (10.0)	748,934 (10.7)	526,396 (9.3)	0.05	8,619 (11.0)	6,203 (11.2)	2,416 (10.4)	0.03
>70 to ≤80	1,096,430 (8.6)	671,164 (9.6)	425,266 (7.5)	0.07	9,650 (12.3)	6,984 (12.6)	2,666 (11.5)	0.03
>80 to ≤90	928,621 (7.3)	584,906 (8.3)	343,715 (6.0)	0.09	11,475 (14.6)	8,413 (15.2)	3,062 (13.2)	0.06
>90 to ≤100	642,510 (5.1)	428,758 (6.1)	213,752 (3.8)	0.11	12,406 (15.8)	9,380 (16.9)	3,026 (13.0)	0.11
Missing	21,716 (0.2)	16,712 (0.2)	5,004 (0.1)	0.04	556 (0.7)	483 (0.9)	73 (0.3)	0.07
**HHS Region** ^††^								
1	796,402 (6.3)	352,686 (5.0)	443,716 (7.8)	0.11	3,196 (4.1)	1,923 (3.5)	1,273 (5.5)	0.10
2	1,129,459 (8.9)	616,638 (8.8)	512,821 (9.0)	0.01	7,203 (9.2)	5,206 (9.4)	1,997 (8.6)	0.03
3	1,472,982 (11.6)	772,703 (11.0)	700,279 (12.3)	0.04	8,135 (10.3)	5,454 (9.8)	2,681 (11.5)	0.05
4	2,542,060 (20.0)	1,587,467 (22.6)	954,593 (16.8)	0.15	16,108 (20.5)	12,412 (22.4)	3,696 (15.9)	0.17
5	2,143,140 (16.9)	1,084,671 (15.4)	1,058,469 (18.6)	0.08	11,893 (15.1)	7,446 (13.4)	4,447 (19.1)	0.15
6	1,253,738 (9.9)	793,820 (11.3)	459,918 (8.1)	0.11	10,517 (13.4)	7,726 (13.9)	2,791 (12.0)	0.06
7	676,509 (5.3)	346,395 (4.9)	330,114 (5.8)	0.04	2,806 (3.6)	1,873 (3.4)	933 (4.0)	0.03
8	433,653 (3.4)	231,531 (3.3)	202,122 (3.6)	0.01	1,694 (2.2)	1,073 (1.9)	621 (2.7)	0.05
9	1,608,310 (12.7)	885,149 (12.6)	723,161 (12.7)	0	13,495 (17.2)	9,758 (17.6)	3,737 (16.1)	0.04
10	585,995 (4.6)	311,662 (4.4)	274,333 (4.8)	0.02	2,745 (3.5)	1,882 (3.4)	863 (3.7)	0.02
Missing	63,928 (0.5)	40,246 (0.6)	23,682 (0.4)	0.02	826 (1.1)	636 (1.1)	190 (0.8)	0.03
**Rural/Urban classification^§§^**								
Rural	2,566,503 (20.2)	1,581,607 (22.5)	984,896 (17.3)	0.13	14,092 (17.9)	10,475 (18.9)	3,617 (15.6)	0.09
Urban	10,139,673 (79.8)	5,441,361 (77.5)	4,698,312 (82.7)	0.13	64,526 (82.1)	44,914 (81.1)	19,612 (84.4)	0.09
**Respiratory disease**								
Asthma	904,139 (7.1)	478,798 (6.8)	425,341 (7.5)	0.03	7,680 (9.8)	5,393 (9.7)	2,287 (9.8)	0
COPD	1,377,175 (10.8)	825,581 (11.8)	551,594 (9.7)	0.07	15,652 (19.9)	11,066 (20.0)	4,586 (19.7)	0.01
Other chronic lung disease	863,446 (6.8)	507,600 (7.2)	355,846 (6.3)	0.04	24,094 (30.6)	17,396 (31.4)	6,698 (28.8)	0.06
**Cardiovascular disease**								
Heart failure	1,099,317 (8.7)	665,610 (9.5)	433,707 (7.6)	0.07	36,985 (47.0)	26,223 (47.3)	10,762 (46.3)	0.02
Ischemic heart disease	2,689,861 (21.2)	1,529,106 (21.8)	1,160,755 (20.4)	0.03	39,285 (50.0)	27,497 (49.6)	11,788 (50.7)	0.02
Hypertension	8,765,015 (69.0)	4,893,880 (69.7)	3,871,135 (68.1)	0.03	68,951 (87.7)	48,479 (87.5)	20,472 (88.1)	0.02
Other	4,465,517 (35.1)	2,499,783 (35.6)	1,965,734 (34.6)	0.02	56,368 (71.7)	39,466 (71.3)	16,902 (72.8)	0.03
**Cerebrovascular disease**								
Stroke	412,153 (3.2)	248,517 (3.5)	163,636 (2.9)	0.04	6,978 (8.9)	5,091 (9.2)	1,887 (8.1)	0.04
Other	201,661 (1.6)	125,875 (1.8)	75,786 (1.3)	0.04	4,447 (5.7)	3,230 (5.8)	1,217 (5.2)	0.03
**HIV**								
HIV Infection**^¶¶^**	14,740 (0.1)	6,814 (0.1)	7,926 (0.1)	0.01	915 (1.2)	627 (1.1)	288 (1.2)	0.01
**Neurologic and musculoskeletal disease**								
Dementia (including Alzheimer disease)	613,704 (4.8)	397,074 (5.7)	216,630 (3.8)	0.09	4,563 (5.8)	3,407 (6.2)	1,156 (5.0)	0.05
Other	2,696,976 (21.2)	1,532,313 (21.8)	1,164,663 (20.5)	0.03	28,662 (36.5)	20,407 (36.8)	8,255 (35.5)	0.03
**Mental health condition**								
Depression	1,883,167 (14.8)	1,044,477 (14.9)	838,690 (14.8)	0	15,605 (19.8)	10,960 (19.8)	4,645 (20.0)	0.01
**Hematologic disease**								
Blood disorders	364,164 (2.9)	203,907 (2.9)	160,257 (2.8)	0.01	10,696 (13.6)	7,585 (13.7)	3,111 (13.4)	0.01
**Endocrine or metabolic disease**								
Diabetes type I	135,667 (1.1)	75,941 (1.1)	59,726 (1.1)	0	8,895 (11.3)	6,423 (11.6)	2,472 (10.6)	0.03
Diabetes type II	3,297,417 (26.0)	1,921,942 (27.4)	1,375,475 (24.2)	0.07	56,024 (71.3)	39,574 (71.4)	16,450 (70.8)	0.01
Diabetes due to underlying condition or other specified diabetes	189,711 (1.5)	110,823 (1.6)	78,888 (1.4)	0.02	9,430 (12.0)	6,841 (12.4)	2,589 (11.1)	0.04
Other	9,927,282 (78.1)	5,389,421 (76.7)	4,537,861 (79.8)	0.08	71,473 (90.9)	50,227 (90.7)	21,246 (91.5)	0.03
**Gastrointestinal and hepatic disease**								
Chronic liver disease	690,767 (5.4)	386,244 (5.5)	304,523 (5.4)	0.01	10,979 (14.0)	7,800 (14.1)	3,179 (13.7)	0.01
**Obesity**								
Clinical obesity	2,296,440 (18.1)	1,280,953 (18.2)	1,015,487 (17.9)	0.01	26,447 (33.6)	18,654 (33.7)	7,793 (33.5)	0
**Disability status**								
Disabled	1,640,013 (12.9)	878,602 (12.5)	761,411 (13.4)	0.03	15,708 (20.0)	11,099 (20.0)	4,609 (19.8)	0
**Influenza vaccination status**								
Received 2021–22 flu vaccine	8,505,872 (66.9)	3,853,154 (54.9)	4,652,718 (81.9)	0.61	66,330 (84.4)	45,489 (82.1)	20,841 (89.7)	0.22
Received 2022–23 flu vaccine	8,460,188 (66.6)	3,492,915 (49.7)	4,967,273 (87.4)	0.89	64,918 (82.6)	43,480 (78.5)	21,438 (92.3)	0.40
Received 2022–23 flu vaccine and COVID-19 vaccine on same date	1,924,540 (15.1)	0 (—)	1,924,540 (33.9)	1.01	2,280 (2.9)	0 (—)	2,280 (9.8)	0.47
**Original monovalent COVID-19 booster vaccine status**								
Received***	10,540, 003 (83.0)	5,089,503 (72.5)	5,450,500 (95.9)	0.68	61,680 (78.5)	40,733 (73.5)	20,947 (90.2)	0.44

**Abbreviations:** COPD = chronic obstructive pulmonary disease; ESRD = end stage renal disease; flu = influenza; HHS = U.S. Department of Health and Human Services; MS = musculoskeletal; NH = non-Hispanic; SMD = standardized mean or proportion difference.

* Defined as having at least one dialysis encounter (excluding acute kidney injury) in the 90 days preceding the index date. Persons with ESRD receiving dialysis are eligible for Medicare benefits, regardless of age.

^†^ Beneficiaries had documented claims for ≥2 original monovalent mRNA vaccine doses, ≥2 Novavax vaccine doses, or ≥1 Janssen vaccine dose. A single dose (i.e., Janssen), second dose, third dose, or monovalent booster administration code was considered adequate to meet inclusion criteria.

^§^ Defined as receipt of a bivalent mRNA COVID-19 vaccine dose at least 7 days earlier or receipt of original monovalent doses only. Bivalent doses were identified using codes from the Healthcare Common Procedure Coding System and Current Procedural Terminology and must have been administered after August 31, 2022. Beneficiaries’ vaccination status could change during the study period.

^¶^ A standardized mean difference of ≤0.1 indicates a negligible difference in means or proportions between groups.

** https://www.atsdr.cdc.gov/placeandhealth/svi/index.html

^††^ https://www.hhs.gov/about/agencies/iea/regional-offices/index.html

^§§^ https://www.cdc.gov/nchs/data_access/urban_rural.htm#Use_of_the_Urban-Rural_Classification_with_Natality_and_Mortality_Files

**^¶¶^** Defined as ≥2 encounters with *International Classification of Diseases, Tenth Revision, Clinical Modification* code consistent with HIV diagnosis within 183 days before index date.

*** Documentation of third dose or original monovalent vaccine booster administration code. Because there was documentation of receipt of original monovalent booster doses after the index date for some beneficiaries, this variable was considered time-varying. Data presented reflect status as of censoring date.

Among 78,618 Medicare beneficiaries aged ≥18 years with ESRD receiving dialysis who did not have additional immunocompromising conditions and had previously received original COVID-19 vaccine, 23,229 (29.5%) received a bivalent dose, including 7,239 (31.2%) aged 18–64 years and 15,990 (68.8%) aged ≥65 years. Similar to beneficiaries aged ≥65 years, among recipients with ESRD receiving dialysis, a higher percentage of those who received a bivalent vaccine dose compared with those who had not, had also received an influenza vaccine during the 2021–22 season (90% versus 82%) and the 2022–23 season (92% versus 79%) and had received an original monovalent booster vaccine dose (90% versus 74%). In addition, a higher percentage of bivalent COVID-19–vaccinated ESRD beneficiaries were older (69% were aged ≥65 years) and non-Hispanic White (53%) compared with those who did not receive the bivalent COVID-19 vaccine (59% and 47%, respectively).

### Vaccine Effectiveness in Preventing COVID-19–related Thromboembolic Events

During the study period, COVID-19–related thromboembolic events were recorded among 22,001 immunocompetent beneficiaries aged ≥65 years and 1,040 immunocompetent beneficiaries aged ≥18 years with ESRD receiving dialysis (Table 2). A total of 1,505,533,898 original-vaccine–only person-days were contributed by immunocompetent beneficiaries aged ≥65 years, during which 17,746 COVID-19–related thromboembolic events were identified (Table 3). Among adults aged ≥65 years, 694,184,995 bivalent-vaccine person-days were contributed, during which 4,255 COVID-19–related thromboembolic events were identified. Adjusted VE against COVID-19–related thromboembolic events among immunocompetent beneficiaries aged ≥65 years was 47%, with lower VE estimates ≥60 days after bivalent vaccine receipt (42%) compared with VE estimates 7–59 days after bivalent vaccine receipt (54%).

**TABLE 2:** Summary of COVID-19–related thromboembolic events* among Medicare fee-for-service beneficiaries aged ≥65 years and beneficiaries aged ≥18 years with end stage renal disease receiving dialysis,^†^ by immunocompromise status, age group, and event type — United States, September 2022–March 2023

Age group/Event type^§^	Beneficiaries by age group, ESRD, and immunocompromise status, No. (%)			
	Beneficiaries aged ≥65 yrs		Beneficiaries aged ≥18 yrs with ESRD receiving dialysis	
	Immunocompetent	With immunocompromise^¶^	Immunocompetent	With immunocompromise
**≥18 yrs**				
**Total no. of persons**	**—**	**—**	78,618 (100)	22,391 (100)
Any thromboembolic event	**—**	**—**	1,040 (1.32)	365 (1.63)
Ischemic stroke	**—**	**—**	308 (0.39)	102 (0.46)
Myocardial infarction	**—**	**—**	650 (0.83)	230 (1.03)
Venous thromboembolism	**—**	**—**	82 (0.1)	33 (0.15)
**18–64 yrs**				
**Total no. of persons**	**—**	**—**	30,240 (100)	7,349 (100)
Any thromboembolic event	**—**	**—**	275 (0.91)	87 (1.18)
Ischemic stroke	**—**	**—**	93 (0.31)	26 (0.35)
Myocardial infarction	**—**	**—**	155 (0.51)	49 (0.67)
Venous thromboembolism	**—**	**—**	27 (0.09)	12 (0.16)
**≥65 yrs**				
**Total no. of persons**	12,706,176 (100)	2,346,581 (100)	48,378 (100)	15,042 (67.18)
Any thromboembolic event	22,001 (0.17)	7,432 (0.32)	765 (1.58)	278 (1.85)
Ischemic stroke	8,382 (0.07)	2,316 (0.1)	215 (0.44)	76 (0.51)
Myocardial infarction	10,339 (0.08)	3,627 (0.15)	495 (1.02)	181 (1.2)
Venous thromboembolism	3,280 (0.03)	1,489 (0.06)	55 (0.11)	21 (0.14)

**Abbreviation:** ESRD = end stage renal disease.

* Defined as the first occurrence of clotting outcomes (i.e., myocardial infarction, ischemic stroke, or venous thromboembolism) after index date and 7 days before to 30 days after COVID-19 diagnosis.

^†^ Defined as having at least one dialysis encounter (excluding acute kidney injury) in the 90 days before the index date. Persons with end stage renal disease receiving dialysis are eligible for Medicare benefits, regardless of age.

^§^ Individual thromboembolic events are mutually exclusive. If two thromboembolic events occurred on the same day, the following hierarchy is applied: 1) venous thromboembolism, 2) ischemic stroke, 3) myocardial infarction.

^¶^ Defined as at least two encounters within 183 days before the index date for one or more of the following conditions: hematologic malignancy, other intrinsic immune conditions or immunodeficiency, solid malignancy, transplant, or rheumatologic/inflammatory disorders.

**TABLE 3:** Vaccine effectiveness* of bivalent compared with original monovalent vaccination against COVID-19–related thromboembolic events^†^ among immunocompetent Medicare fee-for-service beneficiaries aged ≥65 years and beneficiaries aged ≥18 years with end stage renal disease receiving dialysis^§^ without additional immunocompromising conditions, by age group and time since vaccination — September 2022–March 2023

Age group/Vaccination status	Immunocompetent beneficiaries aged ≥65 years					Beneficiaries aged ≥18 years with ESRD receiving dialysis without additional immunocompromising conditions				
	No. of beneficiaries	No. of COVID-19–related TE	Total no. of person-days	Median follow-up days contributed to category^¶^	aVE (95% CI)**	No. of beneficiaries	No. of COVID-19–related TE	Total no. of person-days	Median follow-up days contributed to category	aVE (95% CI)^††^
**≥18 yrs**										
Original vaccine only (Ref)^§§^	—	—	—	—	—	55,389	917	10,395,534	181	Ref
Bivalent vaccine overall^¶¶^	—	—	—	—	—	23,229	123	2,394,731	114	51 (39–60)
7–59 days since vaccination	—	—	—	—	—	2,822	53	1,165,617	53	56 (40–68)
≥60 days since vaccination	—	—	—	—	—	20,407	70	1,229,114	61	45 (28–58)
**18–64 yrs**										
Original vaccine only (Ref)	—	—	—	—	—	23,001	255	4,215,882	181	Ref
Bivalent vaccine overall^¶¶^	—	—	—	—	—	7,239	20	694,039	101	56 (33–71)
7–59 days since vaccination	—	—	—	—	—	—	—	—	—	—
≥60 days since vaccination	—	—	—	—	—	—	—	—	—	—
**≥65 yrs**										
Original vaccine only (Ref)	7,022,968	17,746	1,505,533,898	181	Ref	32,388	662	6,179,652	181	Ref
Bivalent vaccine overall^¶¶^	5,683,208	4,255	694,184,995	130	47 (45–49)	15,990	103	1,700,692	116	49 (35–60)
7–59 days since vaccination	350,021	1,492	294,516,234	53	54 (51–56)	1,768	45	806,703	53	52 (32–66)
≥60 days since vaccination	5,333,187	2,763	399,668,761	77	42 (39–45)	14,222	58	893,989	63	43 (23–58)

**Abbreviations:** aVE = adjusted vaccine effectiveness; ESRD = end stage renal disease; Ref = referent group; TE = thromboembolic events.

* Vaccine effectiveness was calculated as (1 − hazard ratio) x 100%.

^†^ Defined as the first occurrence of clotting outcomes (i.e., myocardial infarction, ischemic stroke, or venous thromboembolism) after index date and 7 days before to 30 days after COVID-19 diagnosis.

^§^ Defined as having at least one dialysis encounter (excluding acute kidney injury) in the 90 days preceding the index date. Persons with ESRD receiving dialysis are eligible for Medicare benefits, regardless of age.

^¶^ A single beneficiary can contribute follow-up time in multiple categories. The maximum number of post-bivalent vaccination follow-up days = 181.

** aVE was estimated using a doubly robust approach: implementing inverse probability of treatment weighting and further adjusting for adjusting for influenza vaccination status, receipt of original monovalent booster, time since original monovalent vaccine >150 days, and urban/rural residence.

**^††^** aVE was estimated using a doubly robust approach: implementing inverse probability of treatment weighting and further adjusting for age, race, receipt of original monovalent booster, and time since original monovalent vaccine >150 days.

^§§^ Beneficiaries had documented claims for ≥2 original monovalent mRNA vaccine doses, ≥2 Novavax vaccine doses, or ≥1 Janssen vaccine dose. A single dose (i.e., Janssen), second dose, third dose, or monovalent booster administration code was considered adequate to meet the inclusion criteria.

^¶¶^ Defined as receipt of a COVID-19 bivalent mRNA vaccine dose at least 7 days earlier or receipt of original monovalent doses only. Bivalent doses were identified using codes from the Healthcare Common Procedure Coding System and Current Procedural Terminology and must have been administered after August 31, 2022. Beneficiaries could change vaccination status during the study period.

Similarly, a total of 10,395,534 original-vaccine-only person-days were contributed by beneficiaries aged ≥18 years with ESRD receiving dialysis, during which 917 COVID-19–related thromboembolic events were identified. A total of 2,394,731 bivalent vaccine person-days were contributed, during which 123 COVID-19–related thromboembolic events were identified. Adjusted VE against COVID-19–related thromboembolic events was 51%, with lower VE estimates ≥60 days after bivalent vaccine receipt (45%) than 7–59 days after bivalent vaccine receipt (56%); however, these differences were not statistically significant (i.e., the 95% CIs overlapped).

Similar results were seen among beneficiaries aged ≥65 years with immunocompromise (overall bivalent VE = 46%, with 55% VE 7–59 days after receipt of vaccine, and 39% VE ≥60 days post-vaccination) and among beneficiaries with ESRD receiving dialysis and who had additional immunocompromising conditions (overall bivalent VE = 45%, with 60% VE 7–59 days after receipt of vaccine, and nonsignificant 30% VE at ≥60 days post-vaccination) (Supplementary Table 1; https://stacks.cdc.gov/view/cdc/140316). A supplementary analysis estimating VE against all-cause thromboembolic events also indicated a protective effect of bivalent vaccination (Supplementary Table 2; https://stacks.cdc.gov/view/cdc/140315).

## Discussion

During September 4, 2022–March 4, 2023, effectiveness of a bivalent COVID-19 vaccine compared with receipt of original monovalent vaccine alone against COVID-19–related thromboembolic events was 47% among Medicare beneficiaries aged ≥65 years and 51% among Medicare beneficiaries aged ≥18 years with ESRD receiving dialysis. These findings can be interpreted as the incremental benefit of a recent bivalent dose compared with earlier receipt of original monovalent doses and are consistent with reported lower rates of COVID-19–related thromboembolic events among vaccinated than among unvaccinated persons (*5*).

### Context to Risk-Benefit Considerations

The findings that bivalent COVID-19 vaccine provided protection against COVID-19–related thromboembolic events are important considering a January 13, 2023, joint statement**** by CDC and the Food and Drug Administration regarding a rapid-response investigation of a preliminary safety signal detected in the Vaccine Safety Datalink (VSD), a vaccine safety monitoring system. The signal was detected in a vaccinated concurrent comparator analysis and raised a question about whether receipt of a Pfizer-BioNTech bivalent COVID-19 mRNA vaccine increased the risk for an ischemic stroke event in the 21 days following vaccination in persons aged ≥65 years. As additional data accumulated in VSD in early 2023, the signal attenuated and was no longer statistically significant; review of additional studies have not provided clear and consistent evidence of a safety problem with ischemic stroke and bivalent mRNA COVID-19 vaccines.^††††^ Factors other than vaccination, such as unmeasured confounding or selection bias, might have contributed to the VSD signal. The findings in this report provide important context to risk-benefit considerations and highlight the protective effect of bivalent COVID-19 vaccination against COVID-19–related thromboembolic events among adults aged ≥65 years and among adults aged ≥18 years with ESRD receiving dialysis. The supplementary analysis estimating VE against all-cause thromboembolic events, irrespective of COVID-19 diagnosis, also indicated a protective effect of bivalent vaccination. Persons with ESRD receiving dialysis are at high risk for thromboembolic events (*6*). The findings in this report suggest that recent receipt of a COVID-19 bivalent vaccine dose was protective against COVID-19–related thromboembolic events among this high-risk population.

### Duration of Protection

In this analysis, protection afforded by a bivalent dose against COVID-19–related thromboembolic events appeared to wane, with VE decreasing over time since the last dose. However, these results should be interpreted with caution, as only two periods since last dose were assessed in this study. Furthermore, VE estimates by time since dose among beneficiaries with ESRD receiving dialysis did not differ substantially. Previous CDC studies have shown that VE against COVID-19–associated hospitalization wanes, but more durable protection against critical illness (i.e., intensive care unit admission or death), persists for up to 179 days postvaccination (*4*).

### Limitations

The findings in this study are subject to at least five limitations. First, the results of this analysis should be interpreted in the context of underlying immunity as the incremental benefit provided by COVID-19 vaccination. Because of underascertainment of COVID-19 vaccine receipt in medical claims data during the early period of vaccine distribution, assessing absolute VE (i.e., comparing vaccinated and unvaccinated persons) was not possible. Models were adjusted for previous COVID-19 illness reported through Medicare fee-for-service claims data; however, the analysis cannot account for previous SARS-CoV-2 infection among persons without medical encounters. According to a national seroprevalence survey, a large proportion of the population has now experienced SARS-CoV-2 infection (>70% by the third quarter of 2022)^§§§§^; infection-induced immunity decreases the risk for future medically attended COVID-19 illness and might affect observed VE against COVID-19–related thromboembolic events. Second, because of timing of COVID-19 vaccine policy implementation (*7*), this analysis compared recent receipt of a bivalent dose with earlier receipt of an original monovalent vaccine dose. Thus, a direct comparison between bivalent doses and original vaccine doses by similar time since dose was not feasible within the same calendar period. Third, although models were adjusted for relevant confounders such as age and calendar time, residual confounding is possible, including by behavioral differences, history of previous SARS-CoV-2 infection not requiring a medical encounter, history of COVID-19 illness >365 days before the index date, and use of COVID-19 treatments such as nirmatrelvir-ritonavir (Paxlovid). Fourth, COVID-19–related thromboembolic events were ascertained using medical claims data, which might have limitations compared with imaging or other diagnostic test results (*8*). COVID-19–related thromboembolic events in this analysis were limited to events recorded in the inpatient setting to reduce likelihood of misclassification. Finally, because only Medicare beneficiaries enrolled in Part A (hospital insurance) and Part B (medical insurance) are included, the results of this analysis might not be representative of the entire U.S. population aged ≥65 years or all persons aged ≥18 years with ESRD receiving dialysis.

### Implications for Public Health Practice

Among adults aged ≥65 years, a recent bivalent mRNA COVID-19 vaccine dose helped provide protection against COVID-19–related thromboembolic events compared with more distant receipt of original monovalent doses alone. This pattern of protection was also observed among adults with ESRD receiving dialysis, a population particularly susceptible to thromboembolic events. To prevent COVID-19–related complications, including thromboembolic events, adults should stay up to date with recommended COVID-19 vaccination (*9*).

## Footnotes

* Defined as having at least one dialysis encounter (excluding acute kidney injury) in the 90 days before the index date. Persons with ESRD receiving dialysis are eligible for Medicare benefits, regardless of age.

† Eligible beneficiaries were continuously enrolled in Medicare Parts A and B but not part C for at least 365 days before the index date and were eligible to receive a bivalent mRNA COVID-19 vaccine dose. In addition, beneficiaries must not have received a kidney transplant (ESRD cohort), dialysis encounter (Medicare beneficiaries aged ≥65 years cohort), hospice care, or COVID-19 monoclonal antibody treatment within 90 days of the index date, resided in a nursing home consecutively for ≥100 days within 365 days of the index date, or had a COVID-19 diagnosis within 30 days of index date.

§ Defined as receipt of a bivalent mRNA COVID-19 vaccine dose at least 7 days earlier or receipt of original monovalent doses only. Bivalent doses were identified using codes from the Healthcare Common Procedure Coding System and Current Procedural Terminology and must have been administered after August 31, 2022. Beneficiaries could change vaccination status during the study period.

¶ Covariates included demographics (age, sex, race, Social Vulnerability Index, and state and rural/urban classification) and underlying medical conditions. Underlying medical conditions were treated as binary variables and required at least one encounter with the appropriate *International Classification of Diseases, Tenth Revision* code within 365 days from the index date. Time-varying covariates included receipt of an original monovalent booster dose, whether time since last COVID-19 vaccine dose was >150 days, receipt of monoclonal antibody or antiviral treatment, and previous medical claims listing a COVID-19 diagnosis.

** COVID-19–related thromboembolic events were defined as the first occurrence of such events in the inpatient setting after the index date and 7 days before to 30 days after COVID-19 diagnosis. Occurrence of myocardial infarction or ischemic stroke was defined as the presence of a diagnosis code in any position on an inpatient claim; venous thromboembolism was defined as a venous thromboembolism diagnosis in any position on an inpatient claim reported as present on admission, combined with a relevant procedure code in any claim setting within 7 days before or after admission date. COVID-19–related thromboembolic events occurring in the first 7 days after vaccination were not counted. A supplementary analysis considered all-cause thromboembolic events, regardless of relation to COVID-19.

†† Because many COVID-19 primary vaccination series doses among Medicare beneficiaries were administered at mass vaccination clinics where Medicare claims might not be filed, determining whether beneficiaries were in fact unvaccinated was not possible. Thus, this study was limited to beneficiaries with documented evidence of receipt of original COVID-19 vaccine doses.

§§ Beneficiaries had documented claims for ≥2 original monovalent mRNA vaccine doses, ≥2 Novavax vaccine doses, or ≥1 Janssen vaccine dose. A single dose (i.e., Janssen), second dose, third dose, or monovalent booster administration code was considered adequate to meet the inclusion criteria.

¶¶ Follow-up continued until the earliest occurrence of an outcome, death, disenrollment in Medicare Parts A or B, enrollment in Medicare Part C, a nursing home stay lasting ≥100 days or admission to a hospice facility, a dialysis encounter (aged ≥65 years cohort) or a kidney transplant (ESRD cohort), receipt of multiple bivalent booster doses or a dose received <60 days from the last COVID-19 vaccine dose, or end of study period.

*** To adjust for confounders between the bivalent and original-only cohorts, inverse probability of treatment weights was estimated using a proportional hazards model to estimate the propensity for receiving a bivalent dose based on covariates. A marginal structural Cox model estimated the hazard ratio and 95% CIs among the bivalent cohort versus the original cohort, using a doubly robust approach: implementing inverse probability treatment weights and adjusting for influenza vaccination status, receipt of original monovalent booster, whether time since original monovalent vaccine was >150 days, and urban/rural residence (aged ≥65 years cohort) and adjusting for age, race, receipt of original monovalent booster, and time since original monovalent vaccine >150 days (ESRD cohort).

††† Vaccine effectiveness was calculated as (1 – hazard ratio) × 100%, where hazard ratio is the estimated hazard ratio comparing bivalent mRNA COVID-19 vaccine recipients to original monovalent-only COVID-19 vaccine recipients.

§§§ Immunocompromise was defined as at least two encounters within 183 days before the index date for one or more of the following conditions: hematologic malignancy, other intrinsic immune conditions or immunodeficiency, solid malignancy, transplant, or rheumatologic/inflammatory disorders. Immunocompetent was defined as absence of immunocompromise. ESRD alone was not considered an immunocompromising condition, as persons with ESRD were not considered to be moderately or severely immunocompromised in COVID-19 vaccine recommendations.

¶¶¶ 45 C.F.R. part 46.102(l)(2), 21 C.F.R. part 56; 42 U.S.C. Sect. 241(d); 5 U.S.C. Sect. 552a; 44 U.S.C. Sect. 3501 et seq.

****
https://www.fda.gov/vaccines-blood-biologics/safety-availability-biologics/cdc-and-fda-identify-preliminary-covid-19-vaccine-safety-signal-persons-aged-65-years-and-older

††††
https://www.cdc.gov/vaccines/acip/meetings/downloads/slides-2023-10-25-26/01-VaxSafety-Shimabukuro-508.pdf

§§§§
https://www.cdc.gov/vaccines/acip/meetings/downloads/slides-2023-04-19/06-COVID-Oliver-508.pdf
